# Pet dogs’ behavior when the owner and an unfamiliar person attend to a faux rival

**DOI:** 10.1371/journal.pone.0194577

**Published:** 2018-04-18

**Authors:** Emanuela Prato-Previde, Velia Nicotra, Annalisa Pelosi, Paola Valsecchi

**Affiliations:** 1 Dipartimento di Fisiopatologia Medico-Chirurgica e dei Trapianti, Università degli Studi di Milano, Milano, Italy; 2 Dipartimento di Medicina e Chirurgia, Università degli Studi di Parma, Parma, Italy; 3 Dipartimento di Scienze Chimiche, della Vita e della Sostenibilità Ambientale, Università degli Studi di Parma, Parma, Italy; University of Missouri Columbia, UNITED STATES

## Abstract

While dog owners ascribe different emotions to their pets, including jealousy, research on secondary emotions in nonhuman animals is very limited and, so far, only one study has investigated jealousy in dogs (*Canis familiaris*). This work explores jealousy in dogs one step further. We conducted two studies adapting a procedure devised to assess jealousy in human infants. In each study 36 adult dogs were exposed to a situation in which their owner and a stranger ignored them while directing positive attention towards three different objects: a book, a puppet and a fake dog (Study 1: furry; Study 2: plastic). Overall, the results of both studies do not provide evidence that the behavioral responses of our dogs were triggered by jealousy: we did not find a clear indication that the fake dogs were perceived as real social rivals, neither the furry nor the plastic one. Indeed, dogs exhibited a higher interest (i.e. look at, interact with) towards the fake dogs, but differences in the behavior towards the fake dog and the puppet only emerged in Study 2. In addition, many of the behaviors (protest, stress, attention seeking, aggression) that are considered distinctive features of jealousy were not expressed or were expressed to a limited extent, revealing that dogs did not actively try to regain their owner’s attention or interfere with the interaction between the owner and the faux rival. Finally, a differentiated response towards the attachment figure (the owner) and the unfamiliar person (the stranger) did not emerge. Differently from what reported in human infants, dogs’ behavior towards the attachment figure and the stranger interacting with the potential competitor (in this case, the fake dog) did not significantly differ: in both studies dogs paid attention to the owner and the stranger manipulating the fake dog to the same extent. In conclusion, we do not exclude that dogs could possess a rudimentary form of jealousy, but we suggest that research on this topic should require the use of a real social interloper (conspecific or human) and more naturalistic procedures.

## Introduction

Scientists generally agree that some emotions (“primary” or “basic”) have a long evolutionary history and can be found across a wide range of vertebrate species, due to their fundamental adaptive value [1–6]. Conversely, the presence of “secondary” or “complex” emotions in nonhuman species is a lively debated topic. Many researchers still assume that these emotions require elaborate cognitive abilities and emerge relatively late in human development. It is also believed that they imply self and interpersonal awareness, which is generally considered a uniquely human characteristic [7]. Nevertheless, the perspective on secondary emotions is changing as evidences deriving from developmental and cross-species studies suggest that at least some secondary emotions, due to their fundamental role in regulating social life, are present in other species. In addition, this view is supported by the presence of specific cortical structures and psychological infrastructures in at least some mammals [3,8–12].

Jealousy is a secondary emotion that appears to have a clear, strong adaptive value in maintaining and protecting social relationships and bonds (i.e. sibling-parent, sexual, friendship), enhancing the individual’s fitness [9,13–14]. In the human psychological literature jealousy has been defined as a context-dependent social emotion that requires a social triangle and arises when one individual perceives that an intruder is threatening an important relationship. It is generally expressed by observable negative affective responses (e.g. fear, anger, sadness) and accompanied by overt behaviors directed at restoring the relationship by reducing the threat represented by the interloper and regaining attention and care from a significant social partner [14,15]. It has been proposed that jealousy could also occur outside conscious awareness [9,16], without the need of cortically mediated cognition [13] and in the absence of complex interpretations of the meaning of the social interaction [17]. Thus, at least a primordial form of jealousy could arise in other animals in specific situations where a significant relationship is threatened. The best evidence that jealousy could have a primordial form derives from the literature on human infants, which shows that jealousy can be exhibited within the first two years of life in specific social situations (see [15,18] for reviews).There are several studies indicating that human infants aged from six to twelve months exhibited more protest behaviors, negative vocalizations and proximity seeking (approach and gaze) when their mother was holding an infant-like doll compared with when she held a book and when a stranger held the doll [19–21]. Taken together, these studies suggest that infants are sensitive to the loss of maternal attention, distinguish between social and non-social objects and do not show an undifferentiated general response to any person or object, but react to potential threats to the relationship with the attachment figure. Thus, jealousy could initially have evolved as a behavioral/emotional strategy to protect material and affective resources within the parent-offspring relationship [22]. The fact that jealousy can be recognizable in cognitively immature infants also suggests that this “secondary” emotion could occur with various degrees of complexity across social species, rather than being an all-or-none phenomenon, suddenly appearing in humans during the 2nd year of life or later. In addition, the adaptive function of jealousy in human infants has important theoretical implications since many nonhuman animals, particularly those living in social groups, could face situations that in principle would evoke jealousy in humans [23]. Comparative studies are needed to better understand the evolutionary emergence and the functions of jealousy and its relation with cognitive abilities.

The domestic dog (*Canis lupus familiaris*) has proved to be an interesting and promising animal model to investigate the evolution of social cognition and in the last 20 years researchers showed that dogs have quite sophisticated socio-cognitive abilities that in some cases parallel those reported for human infants [24–31]. Moreover, a growing body of research is shedding light on their emotional life ([12] for a review), suggesting that, besides primary emotions, dogs could also experience, at least to some extent, more complex emotional states, such as empathic-like responses [32,33] and inequity aversion [34–36]. Recently, Harris & Prouvost [37] suggested that dogs could express jealousy when their owner directs his/her attention to a potential rival, providing some support to people’s belief that animals, especially dogs, show jealousy when attention and affection are given to another person or animal [38–40]. Indeed, dogs are suitable subjects for investigating the existence of jealousy as they: 1. form stable groups and differentiate social relationships with conspecifics [41–42]; 2. establish a strong relationship with their owner, characterized by dependency for physical and psychological resources, which is functionally comparable to an infantile attachment [43]; 3. discriminate human emotions [44–46], are sensitive to others’ attentional states [47–49] and to unfair treatment [34–36]. Hence, comparing dogs and infants, using the same type of procedures and analyses, may help to unravel proximate and ultimate causes of emotional behaviors.

Adapting a paradigm from human infant studies, Harris and Prouvost [37] tested dogs at their homes in three different conditions in which their owner ignored them while affectionately interacting with different objects. In the “jealousy” condition owners petted and sweetly talked to a realistic-looking stuffed dog that barked and wagged its tail; in the second condition, they directed the same behavior to a jack-o-lantern pail; in the third condition, owners were instructed to read aloud a book which played melodies. At the end of each condition, the owner put the object down within the dog’s reach and walked away. It emerged that a significantly higher percentage of dogs showed behaviors that the authors considered indicative of jealousy (pushing/touching the owner and the object, getting between the owner and the object, snapping and whining) when the owner was manipulating the stuffed dog compared to both the jack-o-lantern and the book. These findings represent an interesting starting point to assess jealousy in dogs. However, some methodological issues suggest caution in drawing conclusions: for example, the authors recorded the number of subjects expressing a certain behavior in each condition, but this only provides partial information on the phenomenon, since the robustness of the behavioral response (e.g. duration) is neglected. When they estimated the amount of time (percentage of point samples) spent by dogs attending to the owner (i.e. looking at the owner), no difference emerged between the stuffed dog and the novel object (jack-o-lantern) conditions. Conversely, dogs directed a significantly higher attention (i.e. looking) to the animated stuffed dog compared to both the jack-o-lantern and the book. These results could be due to different emotional/motivational states rather than jealousy: as many pet dogs are familiar with furry and squeaking toys and are used to play with them, it cannot be excluded that they considered the stuffed dog just a toy to play with. Last but not least, in our opinion, since the function of jealousy is to protect a valuable relationship from an intruder, in order to provide more conclusive evidence it would be important to compare the dogs' behavior towards the owner (the attachment figure) and towards an unfamiliar person giving attention to a potential rival. Human infants differentiate among potential rivals (e.g. a book vs. a realistic doll) and also react in different ways when their mother rather than an unfamiliar adult gives positive attention to a potential rival [18].

The current work explores jealousy in dogs one step further, pursuing two goals: 1. clarify whether dogs can really be deceived by a fake dog, considering it a social rival rather than a toy; 2. evaluate whether the identity of the humans involved plays a role in triggering jealousy behaviors.

The dogs were presented with two types of fake dogs differing in appearance and size (Study 1: furry stuffed dog; Study 2: plastic dog), a novel object (a puppet) that did not appear like a conspecific but had some potentially appealing features for a dog (big eyes and soft fabric), and a picture book (a familiar and non social object). The latter was used as a control stimulus to ascertain to what extent dogs would react to the mere loss of people's attention. The identity of the handler of the potential rivals was controlled introducing an unfamiliar person in the testing situation, as done by Hart and colleagues [19] with human infants. Thus, in our testing situation, both the owner and an unfamiliar person interacted with the three different objects, directing positive attention and affection towards them. Assuming that our experimental subjects could be deceived by the fake dogs and therefore be driven to jealousy, we expected they would: 1. sniff the ano-genital region of the fake dog, a behavioral pattern that dogs exhibit during social interactions with conspecifics for individual recognition [37]; 2. show some aggression towards the fake dog to prevent the rival to interfere with the relationship with the owner [37,50]; 3. show higher levels of attention/interaction, vocalizations and stress signals in the fake dog condition compared to the puppet condition to gain their owner attention and to disrupt the interaction [37,19]; 4. show attention/interaction, vocalizations and stress signals only when their owner gave affection to the fake dog, since dogs are not affectionately bonded with the stranger [19]; 5. show a limited behavioral response in the book condition, since the book is just a familiar and non-social object [37,19].

## Material and methods

### Experimental setting and stimuli

Testing took place in two different locations: one at the Canis sapiens Lab of the Università degli Studi di Milano and the other one at the Dipartimento di Scienze Chimiche, della Vita e della Sostenibilità Ambientale of the Università degli Studi di Parma. Testing rooms were not identical but both measured approximately 4.5 x 3.5 m and were equipped with two chairs (one for the stranger and one for the owner) facing each other at a distance of 120 cm, a soft plastic carpet between them, a water bowl and a small table with a computer positioned on it. The computer was used to guide and standardize the owner's and the stranger's actions throughout the procedure: a PowerPoint presentation provided written instructions at predetermined fixed intervals. An HD video camera was placed on a wall in a corner of the room to record the test.

**Fig 1:**
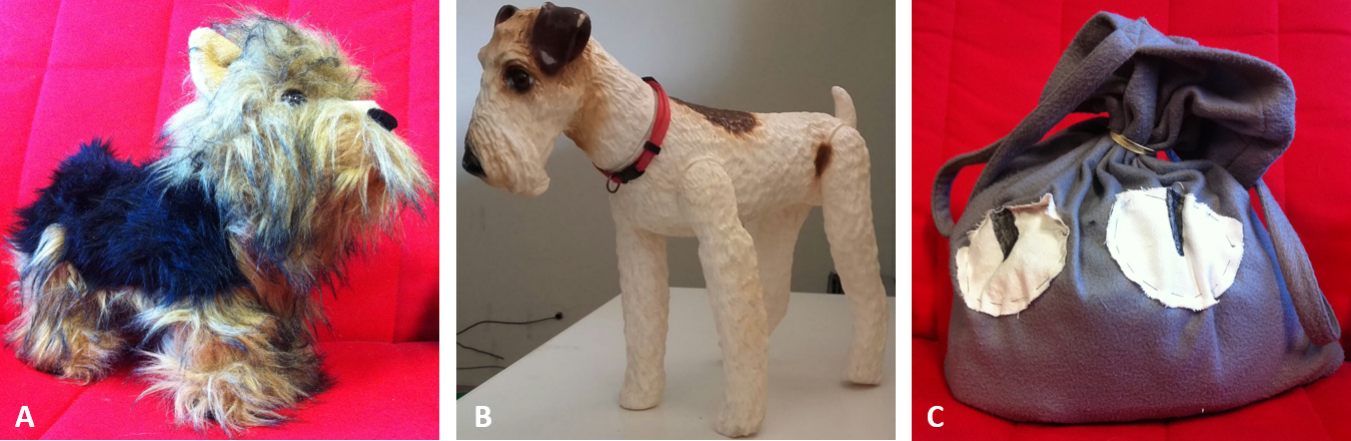
The three objects used in study 1 and 2. A: Furry fake dog; B: Plastic fake dog; C: Puppet.

Three different objects were used during the test: a fake dog, a soft puppet and a picture book. In Study 1 the fake dog was a furry Yorkshire terrier (length 32 cm, height at the withers 18 cm, Fig 1A); in Study 2 the fake dog was a plastic Fox terrier (length 50 cm, height at the withers 30 cm, Fig 1B). In both studies the puppet was a soft hand-made bag with a light grey fleece and two 'eyes' fixed on it (40 x 37 cm, Fig 1C). The objects were all static and did not emit sounds.

Two different female researchers (one in Milano and the other in Parma) played the role of the stranger.

### Experimental design

To test our hypotheses, in both studies we set up a 3 (object type: fake dog, puppet, book) x 2 (object handler: owner, stranger) within-subjects design. Thus, each dog had the opportunity to observe and interact with both the owner and the stranger manipulating the three objects. An a priori power analysis (G*Power 3.1.9.2) for our experimental design (3x2 within-subject) setting alfa = .05, power = .90, an expected medium effect size f = .25, correlation between measures r = .5 and sphericity close to 1 (epsilon = .9), indicated a minimum sample size of 25 subjects.

#### Ethical statement

All procedures were performed in full accordance with Italian legal regulations (National Directive n. 26/14—Directive 2010/63/UE) and the guidelines for the treatments of animals in behavioral research and teaching of the Association for the Study of Animal Behavior (ASAB). The protocol was approved by the Ethical Committee for the Use of Animals (PROT.N. 105/OPBA/2016) of the Università degli Studi di Parma. A written consent to video-record and use data in an anonymous form was obtained by the owners prior to testing.

### Subjects

#### Study 1

Thirty-six healthy adult dogs (15 males, 21 females; 20 pure breed, 16 mixed breed), ranging in age from 1 to 13 years (mean = 5.24 years, SD = 2.74;), and their owners participated in the study. Dog-owner dyads were recruited both by personal contact and from advertisement distributed within the Università di Milano.

Dogs’ inclusion criteria were: being kept exclusively for companionship, living within the human household for at least 1 year, being accustomed to encounter human strangers, being at least 1- year old. Dog-owner dyads were randomly allocated to the different testing sequences and thus we tested 6 dogs for each sequence.

#### Study 2

Thirty-six healthy adult dogs (17 males, 19 females; 19 pure breed, 17 mixed breed), ranging in age from 1 to 10 years (mean = 4.74 years, SD = 2.5), and their owners participated in the study. Dog-owner dyads were recruited both by personal contact and from advertisement distributed within the Università di Parma.

Inclusion criteria were the same as in Study 1. Dog-owner dyads were randomly allocated to the different testing sequences and thus we tested 6 dogs for each sequence.

### Procedure

Researchers met the owner-dog dyads outside the Departments and escorted them to a waiting room were the owner signed a consent form and filled up a questionnaire with information about the dog (age, breed, education and lifestyle). Next, the owner and the dog entered the testing room and the experimenter (a researcher different from the stranger) explained the test procedure to the owner, while the dog was free to explore the room for approximately 10 minutes. The experimenter then left the room. The test started with a 1-min familiarization phase during which the owner initially walked around the room paying attention to some wall posters and then sat on the owner's chair and interacted with his/her dog. The procedure comprised three experimental episodes, each consisting of two 1-min phases during which the owner and the stranger (a researcher unfamiliar to the dog) were both in the room and ignored the dog. In each experimental episode the owner and the stranger alternately handled one of the objects for 1 minute (book, puppet, fake dog) while talking to each other in a friendly manner. For sake of simplicity, the 6 phases will be hereinafter indicated as follows:

FDOW: the fake dog (FD) is handled by the owner (OW);
FDSTR: the fake dog is handled by the stranger (STR);
BOW: the book (B) is handled by the owner;
BSTR: the book is handled by the stranger;
POW: the puppet (P) is handled by the owner;
PSTR: the puppet is handled by the stranger.

These 3 episodes were separated by 1-min intervals identical to the familiarization phase: these intervals aimed at avoiding, or at least reducing, any potential carryover effects from the previous. The order in which the objects were presented during the test and who manipulated them first (i.e. owner or stranger) were partially counterbalanced to avoid order effects on dogs’ behavioral responses. Thus, each dog was exposed to a given sequence of phases and the following six combinations were selected:

1. BSTR-BOW, FDSTR-FDOW, PSTR-POW;
2. BOW-BSTR, FDOW-FDSTR, POW-PSTR;
3. FDSTR-FDOW, PSTR-POW, BSTR-BOW;
4. FDOW-FDSTR, POW-PSTR, BOW-BSTR;
5. PSTR-POW, BTSR-BOW, FDSTR-FDOW;
6. POW-PSTR, BOW-BSTR, FDOW-FDSTR.

At the beginning of each episode, the stranger entered the room holding one of the objects. In combinations 1, 3 and 5, upon entering, she sat on the stranger's chair, manipulating the object that was kept on her lap, and engaged in an affectionate conversation about the object with the owner. In sequences 2, 4, and 6 the stranger, upon entering the room, gave the object to the owner, who manipulated it first keeping it on his/her lap. After 1 minute the object was transferred to the other person, who held it on his/her lap and handled it appropriately, while still conversing The dog was ignored by both the owner and the stranger for the two minutes. At the end of each episode, the stranger left the room, taking the object with her.

### Data collection and analyses

The test was video recorded and analysed using Solomon Coder beta® 15.01.13 (ELTE TTK, Hungary). Behaviors were recorded continuously in terms of duration and frequency of their occurrence according to the ethogram reported in Table 1. The ethogram was developed considering both the literature on human infants [20, 21] and dogs [37, 43] and was refined after a preliminary analysis of the videos to include other potentially interesting behaviors. The ethogram included behaviors directed towards the owner/stranger, the objects and environment and stress related behaviors. Inter-observer agreement was assessed by means of independent parallel coding of a random sample of 16 dogs out of 72 (8 dogs for each experiment; 22.22% of the total number of dogs). The agreement was assessed by Spearman correlation and it was good for all behaviors (Rho ranging from 0.7 to 0.979; Table 1).

**Table 1:** Ethogram used in study 1 and 2 and interobserver agreement.

Category	Pattern	Description	Agreement
**^a^Object Directed Behaviors**	**Chew/bite**	The dog chews or bites the object	Rho = 0.809
	**Interaction with Object**	The dog interacts with the object without ambiguity. This pattern includes behaviors such as touching (with the paws or the muzzle), pushing and sniffing.	Rho = 0.753
	**Look at Object**	The dog looks at the object.	Rho = 0.868
	**Social Investigation**	The dog clearly smells the ano-genital area, the muzzle, or the ears of the fake dog.	Rho = 0.832
**^a^Person Directed Behaviors**	**Attention to Owner**	The dog is oriented with the head and the body towards the owner and can gaze at him/her face.	Rho = 0.858
	**Attention to Stranger**	The dog is oriented with the head and the body towards the stranger, and can gaze at her face.	Rho = 0.874
	**Interaction with Owner**	The dog actively interacts with the owner. This pattern includes behaviors such as being in contact, touching (with the paws or the muzzle), sniffing and jumping on.	Rho = 0.833
	**Interaction with Stranger**	The dog actively interacts with the stranger. This pattern includes behaviors such as being in contact, touching (with the paws or the muzzle), sniffing and jumping on.	Rho = 0.979
**^a^Enviroment Directed Behaviors**	**Explore Room**	The dog explores the room. This pattern includes walking around and careful visual/olfactory exploration.	Rho = 0.929
	**Orientation to Door**	The dog is oriented with the head and the body towards the door, gazing at it.	Rho = 0.762
**^b^Stress Related Behaviors**	**Stress Signals**	Nose-lip licking, shaking, yawning, scratching, stretching, rolling, chewing, raising paw.	Rho = 0.874
**^b^Vocal behavior**	**Vocalizations**	This pattern includes whining and barking.	Rho = 0.937

^a^ behaviors recorded as duration and transformed into percentage of the total time for purpose of statistical analysis

^b^ behaviors recorded as frequency and transformed into percentage of total occurrences for purpose of statistical analysis

Since there was a small variability in the length of each phase, due to owners' differences in readiness to follow the instructions, durations and frequencies were respectively transformed in percentages of the total time and of total occurrences and used as dependent variables in the statistical analysis.

For both studies, the effects of dog’s sex and age, order of the objects presentation (FD-P-B vs. B-FD-P vs. P-B-FD) and order of handlers (OW first vs. STR first) on dogs’ behaviors were evaluated by means of preliminary ANOVAs. No significant effects of any variable emerged.

Repeated measures GLM ANOVAs with object (3 levels: book, fake dog, puppet) and handler identity (2 levels: owner, stranger) as factors, and behaviors as dependent variables were carried out using the Greenhouse-Geisser correction when sphericity assumption was violated. Pairwise post-hoc tests with Bonferroni’s correction were carried out for significant main and interaction effects. All the statistical analyses were carried out with IBM SPSS Statistics 24.

## Results

**Fig 2:**
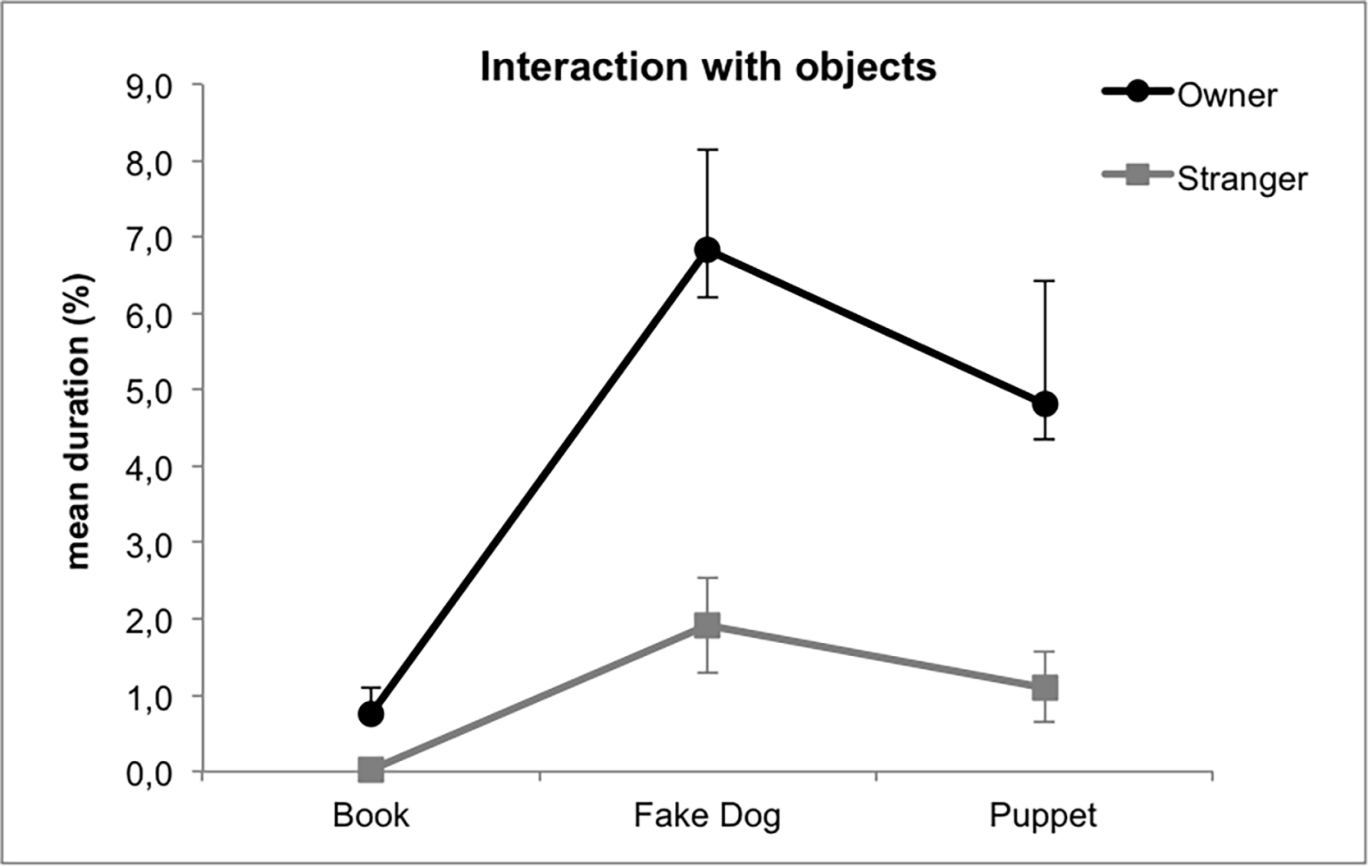
Mean percentage ± SE of time spent by dogs interacting with the object in Study 1. Legend: black line, the owner is manipulating the objects; grey line: the stranger is manipulating the objects. Post-hoc values: Fake dog -Owner vs. Book-Owner, p<0.001; Puppet-Owner vs. Book-Owner, p = 0.035; Fake dog-Stranger vs. Book-Stranger, p = 0.014; Puppet-Stranger vs. Book-Stranger, p = 0.066.

**Fig 3:**
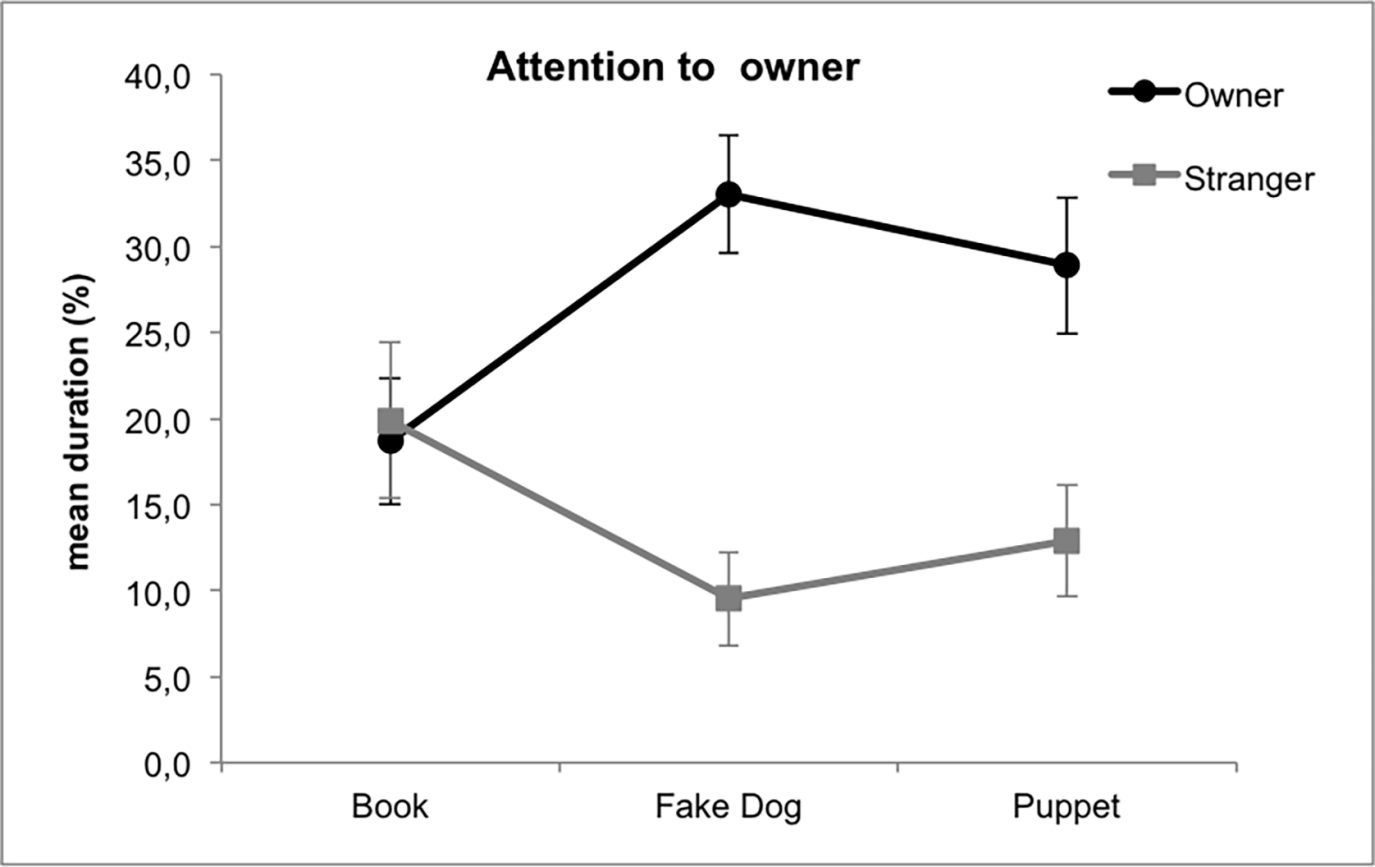
Mean percentage ± SE of time spent by dogs paying attention to the owner in Study 1. Legend: black line, the owner is manipulating the objects; grey line: the stranger is manipulating the objects. Post-hoc values: Fake dog-Owner vs. Fake dog-Stranger, p<0.001; Puppet-Owner vs. Puppet-Stranger, p<0.001; Fake dog-Owner vs. Book-Owner, p = 0.017.

**Fig 4:**
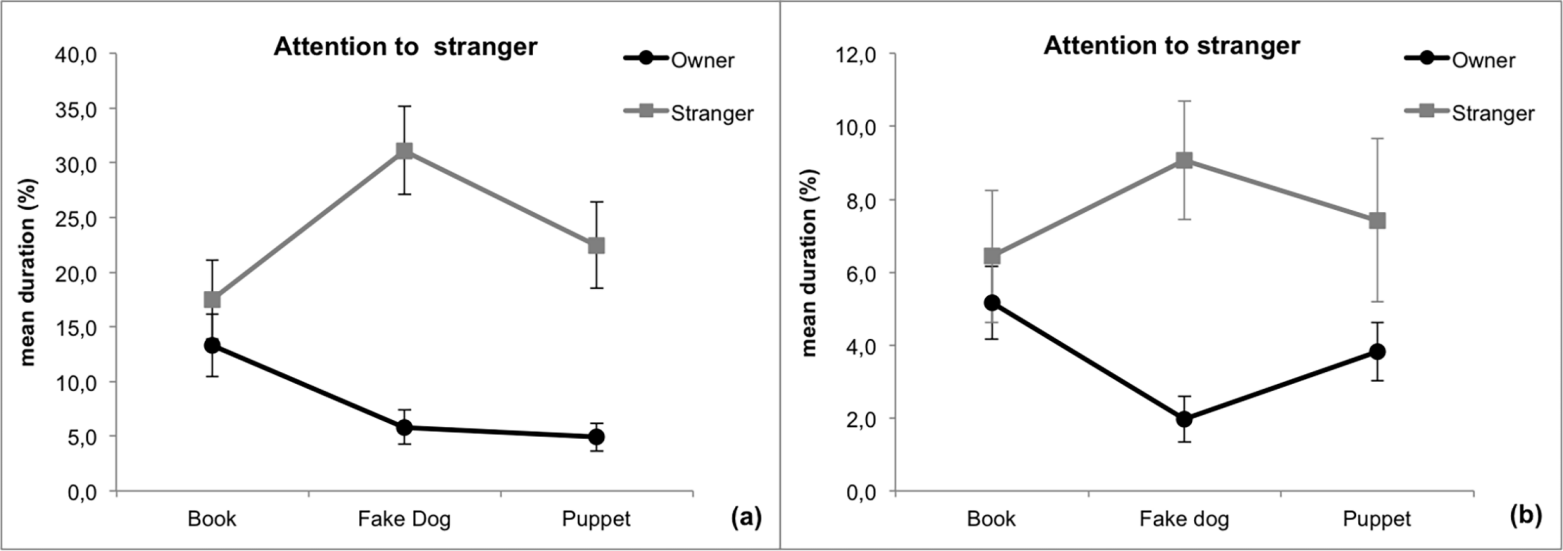
**Mean percentage ± SE of time spent by dogs paying attention to the stranger in Study 1(a) and in Study 2(b)**. Legend: black line, the owner is manipulating the objects; grey line: the stranger is manipulating the objects. Post-hoc values: Fake dog-Stranger vs. Fake dog-Owner, Study 1: p<0.001; Study 2: p<0.001; Puppet-Stranger vs. Puppet-Owner, Study 1: p<0.001; Fake dog-Stranger vs. Book-Stranger, Study 1: p = 0.038.

Data and results of statistical analysis of Studies 1 and 2 are reported in Tables 2–5 and in Figs 2, 3 and 4. In Tables 2 and 4 mean ± SD of each behavior (duration or frequency) are detailed and in Tables 3 and 5 statistical values of the GLM ANOVAs are reported.

**Table 2:** (Study 1). Mean percentage± SD of time spent for each behavior across the experimental phases.

Phase	Interaction with Object	Look at Object	Social investigation	Attention to Owner	Attention to Stranger	Interaction with Owner	Interaction with Stranger	Explore Room	Orientation to Door	Stress
**BOW**	.75±2.06	1.09±2.82	-	18.68±21.99	13.33±17.24	1.399±4.23	1.54±7.03	30.45±25.21	3.84±8.84	2.39±3.10
**FDOW**	6.83±7.95	14.80±13.95	5.96±7.22	33.06±20.60	5.84±9.53	1.63±3.13	1.39±3.86	10.14±17.9	2.10±6.07	2.44±2.85
**POW**	4.81±9.69	4.85±5.77	-	28.89±23.75	4.91±7.57	.75±3.37	1.12±2.71	26.43±25.57	3.72±11.59	2.58±2.59
**BSTR**	0.02±.08	.71±1.81	-	19.89±27.07	17.52±21.74	1.39±4.04	.91±3.26	30.55±28.42	4.75±12.79	2.11±2.15
**FDSTR**	1.92±3.76	12.88±17.77	3.49±5.51	9.52±16.31	31.13±24.02	4.05±15.50	1.53±2.77	16.29±16	1.44±3.47	2.28±3.09
**PSTR**	1.11±2.74	1.49±2.29	-	12.88±19.27	22.46±23.77	4.27±13.79	1.55±3.48	23.18±21.37	3.32±8.79	2.39±2.72

**BOW**: the book is handled by the owner;**FDOW**: the fake dog is handled by the owner; **POW**: the puppet is handled by the owner; **BSTR**: the book is handled by the stranger; **FDSTR**: the fake dog is handled by the stranger; **PSTR**: the puppet is handled by the stranger.

**Table 3:** (Study 1) statistical values of the GLM ANOVA for main and interaction effects.

Effects	Interaction with Object	Look at Object	Social Investigation	Attention to Owner	Attention to Stranger	Interaction with Owner	Interaction with Stranger	Explore Room	Orientation to Door	Stress
**Object**	F_[1.6,58.45]_ = 9.27	F_[1.13,39.6]_ = 35.02		F_[1.59,55.65]_ = .22	F_[1.49,52.21]_ = 1.56	F_[1.385,48.491]_ = 1.22	F_[1.557,54.501]_ = .05	F_[__2__,70]_ = 9.37	F_[1.59,55.79]_ = 1.03	F_[__2__,705]_ = .15
	p = .000	p = .000	-	p = .75	p = .22	p = .219	p = .911	p = .000	p = .36	p = .857
	η_par_^2^ = .21	η_par_^2^ = .50		η_par_^2^ = .006	η_par_^2^ = .04	η_par_^2^ = .034	η_par_^2^ = .002	η_par_^2^ = .21	η_par_^2^ = .03	η_par_^2^ = .004
**Handler**	F_[1,35]_ = 15.03	F_[1,35]_ = 1.78	F_[1,35]_ = 2.93	F_[1,35]_ = 19.78	F_[1,35]_ = 32.58	F_[1,35]_ = 2.266	F_[1,35]_ = .003	F_[1,35]_ = .016	F_[1,35]_ = .003	F[_1,35]_ = .35
	p = .000	p = .19	p = .096	p = .000	p = .000	p = .141	p = .960	p = .69	p = .96	p = .556
	η_par_^2^ = .30	η_par_^2^ = .05	η_par_^2^ = .077	η_par_^2^ = .36	η_par_^2^ = .48	η_par_^2^ = .061	η_par_^2^ = .00	η_par_^2^ = .01	η_par_^2^ = .00	η_par_^2^ = .01
Object*Handler	F_[1.74,63.9]_ = 3.41	F_[1.14, 40.15]_ = .63		F_[2,70]_ = 11.58	F_[2,70]_ = 10.36	F_[1.189,41.615]_ = 1.19	F_[1.505,52.673]_ = .49	F_[2,70]_ = 1.87	F_[1.53,53.44]_ = .31	F[_2,63.01]_ = .014
	p = .046	p = .54	-	p = .000	p = .000	p = .292	p = .561	p = .10	p = .74	p = .986
	η_par_^2^ = .09	η_par_^2^ = .02		η_par_^2^ = .25	η_par_^2^ = .23	η_par_^2^ = .033	η_par_^2^ = .014	η_par_^2^ = .05	η_par_^2^ = .01	η_par_^2^ = .00

**Table 4:** (Study 2): Mean percentage± SD of time spent for each behavior across the experimental phases.

Phase	Interaction with Object	Look at Object	Social investigation	Attention to Owner	Attention to Stranger	Interaction with Owner	Interaction with Stranger	Explore room	Orientation to door	Stress
**BOW**	.60±1.89	1.18±1.76	-	5.74±8.53	5.15±6.02	2.44±7.04	4.68±8.84	36.70±25.08	6.31±14.32	1.11±1.56
**FDOW**	13.40±13.59	14.91±18.57	8.81±11.08	9.78±14.44	1.97±3.77	.91±1.79	2.26±4.26	21.33±21.91	3.60±8.72	1.61±1.99
**POW**	6.31±7.08	6.27±8.22	-	5.96±5.77	3.82±4.81	3.63±10.83	3.11±7.47	34.13±25.09	6.52±17.76	1.19±1.74
**BSTR**	2.13±6.91	.91±1.85	-	7.55±10.54	6.43±10.86	2.08±6.44	6.43±17.70	40.55±31.47	6.10±13.93	1.78±2.34
**FDSTR**	10.04±10.87	11.42±16.05	4.56±6.15	9.60±10.57	9.07±9.72	1.93±4.74	3.86±8.36	28.47±26.26	2.67±7.62	2.06±2.20
**PSTR**	3.42±5.57	2.68±4.41	-	5.87±7.56	7.42±13.47	3.58±11.53	3.15±9.31	34.79±29.24	4.93±8.87	1.5±2.16

**BOW**: the book is handled by the owner; **FDOW**: the fake dog is handled by the owner; **POW**: the puppet is handled by the owner; **BSTR**: the book is handled by the stranger; **FDSTR**: the fake dog is handled by the stranger; **PSTR**: the puppet is handled by the stranger

**Table 5:** (Study 2): statistical values of the GLM ANOVA for main and interaction effects.

Effects	Interaction with Object	Look at Object	Social investigation	Attention to Owner	Attention to Stranger	Interaction with Owner	Interaction with Stranger	Explore Room	Orientation to Door	Stress
**Object**	F_[1.45,50.99]_ = 24.59	F_[1.17,41.15]_ = 17.39		F_[1.691,59.172]_ = 2.26	F_[__2__,70]_ = .015	F_[1.708,59.763]_ = 2.22	F_[1.274,44.588]_ = 1.80	F_[__2__,70]_ = 4.11	F_[1.45,50.74]_ = .99	F_[__2__,70]_ = .99
	p = .000	p = .000	-	p = .121	p = .985	p = .125	p = .186	p = .021	p = .36	p = .374
	η_par_^2^ = .41	η_par_^2^ = .33		η_par_^2^ = .061	η_par_^2^ = .00	η_par_^2^ = .06	η_par_^2^ = .049	η_par_^2^ = .105	η_par_^2^ = .03	η_par_^2^ = .028
**Handler**	F_[__1__,__35__]_ = 1.44	F_[__1__,__35__]_ = 4.43	F_[__1__,__35__]_ = 4.53	F_[__1__,__35__]_ = .18	F_[__1__,__35__]_ = 10.49	F_[__1__,__35__]_ = .041	F_[__1__,__35__]_ = .85	F_[__1__,__35__]_ = 2.01	F_[__1__,__35__]_ = .87	F_[__1__,__35__]_ = 3.85
	p = .238	p = .046	p = .04	p = .671	p = .003	p = .840	p = .361	p = .166	p = .358	p = .058
	η_par_^2^ = .04	η_par_^2^ = .11	η_par_^2^ = .115	η_par_^2^ = .005	η_par_^2^ = .231	η_par_^2^ = .001	η_par_^2^ = .024	η_par_^2^ = .054	η_par_^2^ = .02	η_par_^2^ = .099
Object*Handler	F_[__2__,70]_ = 2.76	F_[1.25,43.85]_ = .91		F_[__2__,70]_ = .42	F_[__2__,70]_ = 3.21	F_[1.336,46.743]_ = .26	F_[__2__,70]_ = .44	F_[__2__,70]_ = .69	F_[1.53,53.44]_ = .13	F_[__2__,70]_ = .25
	p = .07	p = .409	-	p = .660	p = .046	p = .682	p = .643	p = .504	p = .87	p = .782
	η_par_^2^ = .07	η_par_^2^ = .02		η_par_^2^ = .012	η_par_^2^ = .084	η_par_^2^ = .007	η_par_^2^ = .013	η_par_^2^ = .019	η_par_^2^ = .004	η_par_^2^ = .007

### Object directed behaviors

#### Social investigation

In both studies, almost all subjects engaged in Social Investigation (ano-genital region and muzzle) of the fake dogs (Study 1, 30 dogs out of 36 distributed as follow: 12 both in FDOW and FDSTR, 11 only in FDOW and 7 only in FDSTR; Study 2, 28 dogs out of 36 distributed as follow: 16 both in FDOW and FDSTR, 8 only in FDOW and 4 only in FDSTR). Overall, the FD was investigated for about 9.5% of time in Study 1 and 13% of time in Study 2. Time spent investigating the fake dog handled by the owner or the stranger was not significantly different in Study 1 (FDOW vs. FDSTR p = 0.096), while in Study 2 dogs spent a higher amount of time investigating the fake dog in the FDOW than in the FDSTR phase (p = 0.04).

#### Chew/bite

Aggressive bite was never observed, neither in Study 1 nor in Study 2. Dogs sporadically chewed the fake dog and the puppet. In Study 1, 7 dogs out of 36 (19%) chewed the furry fake dog, 5 of them did it in the FDOW phase and 2 both in the FDOW and FDSTR phases. Another dog chewed the puppet only in the POW phase. In Study 2, this behavior was exhibited toward the plastic fake dog and the puppet by only 5 dogs out of 36 (14%): 3 dogs chewed the fake dog and 2 chewed the puppet. No statistical analysis was carried out due to negligible frequency of this behavior.

#### Look at object

In both Study 1 and Study 2 the GLMs revealed a main effect of the object identity: dogs spent significantly more time looking at the fake dog compared to the other objects; the puppet was gazed for a longer time than the book (Study 1: fake dog vs. puppet p<0.001, fake dog vs. book p<0.001, puppet vs. book p = 0.002; Study 2: fake dog vs. puppet p = 0.006, fake dog vs. book p<0.001, puppet vs. book p = 0.001).

#### Interaction with object

In Study 1 (furry fake dog) a significant interaction was found between object and handler identity: overall, dogs interacted significantly longer with the objects when they were manipulated by the owner compared to the stranger (book p = 0.04; fake dog p = 0.001; puppet p = 0.03). As can be seen in Fig 2, dogs interacted longer with the fake dog and with the puppet than with the book, regardless of the identity of the handler (post-hoc values are reported in figure caption).

In Study 2 (plastic fake dog) only the identity of the manipulated object had a significant influence on dogs’ behavior: dogs spent significantly more time interacting with the fake dog compared to the puppet (fake dog vs. puppet, p = 0.001) and to the book (fake dog vs. book p<0.001), and they interacted with the puppet significantly longer than with the book (puppet vs. book p = 0.002).

In sum, in both studies the majority of dogs showed social investigation of the fake dogs, but no aggression towards them, and looked at the fake dog significantly longer than the other two objects. In Study 1, dogs interacted with the furry dog and the puppet to a comparable extent, whereas in Study 2 they interacted with the plastic fake dog significantly longer than with the puppet. Only in Study 1 all objects were more attractive when on the owner's lap.

### Person directed behaviors

#### Attention to owner

In Study 1, the GLM highlighted a significant interaction Object x Handler (Fig 3): in the book condition dogs paid attention to the owner whether or not the book was in his/her hands, whereas in the fake dog and puppet conditions dogs’ attention to the owner was driven by the presence of the object. Furthermore, dogs paid attention to their owner significantly longer when he/she was handling the fake dog compared to the book.

In Study 2, even though dogs addressed their attention to the owner mainly in the FDOW and FDSTR phases, the GLM did not highlight any significant effects of object’s and handler’s identity, nor of the interaction Object x Handler.

#### Attention to stranger

The GLMs revealed a significant interaction Object x Handler in both studies (Fig 4). In Study 1, both the fake dog and the puppet directed the dogs' attention toward the stranger, and dogs paid attention to the stranger for a significantly higher percentage of time when he/she was handling the furry fake dog compared to the book. No other significant differences emerged. In Study 2, only the fake dog directed the dogs' attention toward the stranger.

#### Interaction with owner and stranger

The GLM revealed that physical interaction with the owner and the stranger was not differently affected by the objects and handlers’ identity.

In sum, in both studies dogs oriented their attention/interaction to the owner and to the stranger manipulating the objects to a similar extent. The fake dogs were the more salient stimuli in eliciting these behaviors.

#### Vocalizations and stress related behaviors

Very few dogs tried to attract owner’s attention using vocalizations (Study 1: 8 dogs out of 36, 22%; Study 2: 5 dogs out of 36, 14%), doing it uniformly across the testing phases. No statistical analysis was carried out due to negligible frequency of this behavior.

The frequency of stress related behaviors during testing was low and no statistical differences across testing phases emerged.

Therefore, these behaviors did not provide any useful insight to interpret dogs' behavioral reactions in terms of jealousy.

### Environment directed behaviors

#### Explore room and orienting to door

In both studies the GLMs showed a main effect of the object’s identity on the dogs’ exploratory behavior. Exploration significantly decreased when either the owner or the stranger held the fake dog compared to the other two objects in Study 1, and only compared to the book in Study 2 (Study 1: fake dog vs. book p = 0.002, fake dog vs. puppet p = 0.007, book vs. puppet p = 0.47; Study 2: fake dog vs. book p = 0.016; fake dog vs. puppet p = 0.16; book vs. puppet, p = 1).

As for the time spent being oriented to the door, the GLM did not reveal any difference across the six phases: the dogs spent a similar amount of time oriented to the door whichever objects the owner and the stranger were holding and whoever person was the handler.

## Discussion

The existence of jealousy in dogs, as claimed by Harris and Prouvost [37], is appealing, but experimental evidence is preliminary. The current study aimed at further understanding whether dogs react with a set of behavioral patterns that could indicate a ‘primordial’ form of jealousy when they lose their owner’s attention in favour of a potential rival (a fake dog). For this purpose, we carried out two studies using two types of fake dogs (FD), differing in appearance and texture (Study 1: furry FD; Study 2: plastic FD), a novel object (a puppet) and a book. Moreover, as jealousy should be expressed towards a valuable individual, we controlled for the identity of the handler: thus, the objects were manipulated by the owner and by a stranger who had no social bond with the dog.

Although there were some differences in the behavioral pattern showed by dogs tested in Study 1 and Study 2 (e.g. more attention to people in Study 1 than in Study 2; less interaction with the fake dog in Study 1 than in Study 2; see Tables 2 and 4), results of both studies do not provide convincing proof that the behavioral responses of our dogs were triggered by jealousy, since we did not find clear evidence that the fake dogs were perceived as real social rivals. In addition, dogs did not express, or expressed to a limited extent, many of the behaviors that are considered distinctive features of jealousy (aggression, vocalizations, stress related behaviors, attention/interaction with object and owner). Finally, our dogs did not show, as hypothesized, a differentiated response towards the owner and the stranger.

Our first research question was whether dogs would consider the fake dogs as a real conspecific, as suggested by Harris and Prouvost [37], on the basis of the number of dogs that in their study exhibited social investigation of the fake dog (86%) and aggressive behavior toward it (25%). Even though in our studies 86% and 78% of dogs exhibited social investigation respectively toward the furry and the plastic fake dog, this investigation was a momentary action and not a careful inspection (Furry FD mean duration: FDSTR: 1.99 s; FDOW: 3.47 s; Plastic FD: mean FDSTR: 2.48 s; mean FDOW: 4.63). Although social investigation of the plastic FD occurred significantly longer when it was on owner's lap than on stranger's lap, in our opinion, this result can hardly be considered a convincing proof that the FDs were effective in deceiving adult and experienced dogs. It is worth considering that the FDs did not smell like a real dog and that during intraspecific interactions between dogs social investigation allows for individual/sexual recognition through odour cues [51–52]. Olfaction plays such a pivotal role in dogs' social communication that they can discriminate between a familiar and an unfamiliar person using odor cues [53–54] and, remarkably, they show an asymmetric use of nostril when processing odors—emitted by conspecifics/humans—that differ in terms of emotional valence [55–56]. Moreover, dogs form cross-modal representations of humans and conspecifics integrating auditory and visual stimuli [25, 57]. Likely, a handful of seconds was sufficient for our adult dogs to perceive the FDs as faux and this might account for the complete lack of aggressive behavior. Some of the dogs chewed the FDs, but, differently from what reported by Harris and Prouvost [37], snapping and biting were never observed. It is worth noting that their fake dog was a toy for human infants that barked/whined and wagged its tail, while our FDs were silent and motionless: thus, the aggressive responses they observed could have been motivated by fear of a novel/odd object or by predatory drive rather than by jealousy [58]. It is also possible that the dogs tested at their home by Harris and Prouvost [37] showed aggressive behaviour driven by a protective/territorial motivation [59]. In our opinion a neutral testing environment is more suitable to disentangle aggressive behaviors triggered by jealousy from territorial/protective aggression. However, the choice of the testing location is always critical and presents pros and cons that could be interesting topics for further researches.

If it is reasonable to assume that the FDs are not perceived as real dogs, there are no particular reasons to be jealous: thus, which could be the dogs’ underlying motivation to show interest towards them? As in Harris and Prouvost’s study [37], our dogs preferentially looked at the furry FD than at the soft puppet, but they did not seem to discriminate between them when interacting (sniffing and touching); this suggests that these objects were both perceived as toys, as they were of similar size and of soft texture, and that the furry FD resembled toys most pet dogs are familiar with. Conversely, in Study 2, the FD and the puppet had a different appearance and a different texture and it is possible that dogs prolonged their interaction with the plastic FD to make sense of it.

We also expected that if dogs were jealous they would have shown signs of distress and protest (vocalizations) when attention and care were directed to the FDs, but not to the other objects [37, 60]. Results show that vocalizations were infrequent and distributed across the test phases without any link to a specific object/person. To a certain extent these results are similar to those of Harris and Prouvost, who found that: 1. vocalization were relatively infrequent; 2. there was no difference across conditions in barking; 3. dogs whined more in the jealousy condition than in the book condition, but not the jack-o-lantern condition [37]. Overall, it seems to us that dogs did not bark/whine to regain their owner’s attention and to re-establish the relationship with him/her, but to express frustration for being ignored and for not obtaining full access to a potentially interesting object. Further confirmation derives from the frequency of stress signals, which was low and uniformly distributed across testing phases, indicating an arousal state due to the testing situation. In sum, these findings suggest that the use of the FD as a potential rival in a jealousy evoking paradigm is critical, as the stimulus is unnatural. In the context of temperament assessment, Barnard and colleagues [61] questioned the validity of model devices (a fake dog and a child-like doll): they concluded that adult dogs may perceive these models as social stimuli only at distance and for a few seconds at the beginning of the interaction, since the model does not smell, move or interact like a real conspecific or a real child.

In line with Harris and Provoust’s results [37], the book *per se* was largely overlooked, as dogs spent half of the testing time exploring the room when people handled this object. Although to a lesser extent than in the FD conditions, dogs attended to the owner (and the stranger) also in the book condition. This could have at least two different explanations: 1. being sensitive to human inattentiveness [62]; 2. being uncertain about the situation and looking for human cues [30, 63].

Considering that the adaptive function of jealousy is to protect a valuable relationship from an intruder, it should be expected that an unfamiliar person devoting attention and care to a 'conspecific' would not elicit a jealousy response. Thus, to extend Harris and Prouvost's results [37], we introduced a relevant variation in the experimental paradigm, adopting the procedure devised by Hart and colleagues with human infants [19]. A second person, totally unfamiliar to the dogs (but manipulating the same objects) served as control to exclude that dogs’ reactions were due to a mere interest towards the more salient/desired object or to the frustration of being deprived of an interesting toy. Results of both studies showed that dogs' attention (being oriented and gazing) to the owner and the stranger was driven by the presence of the FDs and the puppet in their lap. On the other hand, their attention was not preferentially directed to the attachment figure (the owner), which in the human literature is considered a fundamental requisite to attribute jealousy to an individual. Differently from our results, and in line with an interpretation in terms of jealousy, Hart and colleagues [19] found that children manifested distress and disapproval of the lack of attention and that these reactions were linked with the identity of the person holding the infant-like doll.

Of course our negative outcome does not necessarily mean that dogs do not possess a rudimentary form of jealousy. Indeed, as highlighted above, we believe that our FDs were inappropriate stimuli to trigger jealousy and rivalry in dogs. It is important to underline that comparing adult dogs and human infants, albeit interesting, requires caution: adult dogs and human infants differ in terms of perceptual abilities, as they rely on different sensory modalities in making sense of the world, in their mobility and possibility to interact with the environment. Physical interaction and contact seeking with the attachment figure are pervasive components of the jealousy response in children [14,15] and also occur in situation of mild distress such as the Strange Situation Test [64]. Nevertheless, these behaviors, which are clearly observable in dogs during the paradigm of separation from the attachment figure [43], were scarcely expressed in our testing conditions, providing further support that the dogs did not perceive the presence of the faux rival as a threat to the relationship with their owner.

In sum, current data does not exclude that dogs may have a rudimentary form of jealousy, but shows that available evidence is still inconclusive and that different experimental paradigms and additional rigorous research is required. For example, replications using a more naturalistic procedure and a real social interloper (either conspecific or human), rather than a false social stimulus, could be more suitable in ascertaining whether dogs experience and show a rudimentary form of jealousy, as strongly believed and carefully described by dog owners [38–39]. Our results also suggest that, as for human infants, the investigation of emotions in nonhuman animals is tricky and hampered by limitations, as behavioural expressions may provide ambiguous information about internal emotional states [12, 22].

## Supporting information

S1 File Breed of dogs participating in study1. (DOCX)

S2 File Breed of dogs participating in study 2. (DOCX)
