# Supplementary material for: Pet dogs’ behavior when the owner and an unfamiliar person attend to a faux rival
Source: PLoS One. 2018 Apr 18;13(4):e0194577. doi: 10.1371/journal.pone.0194577 (PMC5905953; doi:10.1371/journal.pone.0194577)
Supplement: S1 File — (DOCX) [file pone.0194577.s001.docx]

**S1 File. Breed of dogs participating in study 1.**

16 Mixed breed, 2 German shepherd, 2 Miniature Poodle, 1 Bolognese, 2 Fox Terrier, 1 Maltese, 1 German Pinscher, 1 Bichon Frisee, 2 Chihuahua, 1 Dachshund, 1 Irish Red Setter, 1 Beagle, 2 Bernese Mountain Dog, 1 Lagotto Romagnolo, 1 West Highland White Terrier, 1 Siberian Husky.
