# Supplementary material for: Pet dogs’ behavior when the owner and an unfamiliar person attend to a faux rival
Source: PLoS One. 2018 Apr 18;13(4):e0194577. doi: 10.1371/journal.pone.0194577 (PMC5905953; doi:10.1371/journal.pone.0194577)
Supplement: S2 File — (DOCX) [file pone.0194577.s002.docx]

**S2 File. Breed of dogs participating in study 2.**

17 Mixed breed, 2 Jack Russel Terrier, 1 Flat Coated Retriever, 1 German Pinscher, 1 Fox Terrier, 1 Portuguese Water Dog, 1 Volpino Italiano, 1 Petit Basset Griffon Vendéen, 2 Border Collie, 1 Beagle, 1 Bernese Mountain Dog, 1 Labrador Retriever, 1 English Springer Spaniel, 1 Tibetan Terrier, 1 American Pit Bull Terrier, 1 American Staffordshire Terrier, 1 German Shepherd, 1 Golden Retriever
